# Reflections on the use of LLMs for cell annotation

**DOI:** 10.1093/bib/bbag312

**Published:** 2026-06-14

**Authors:** Partha Pratim Ray

**Affiliations:** Department of Computer Applications, Sikkim University, PO Tadong, Gangtok, Sikkim 737102, India

**Keywords:** cell type annotation, biomedical large language models, open-source medical LLMs, single-cell transcriptomics, reproducibility

## Abstract

This letter comments on the recently published AICellType platform for large language model (LLM)-based cell type annotation in single-cell and spatial transcriptomics. While appreciating the authors’ systematic benchmarking and practical contribution, concerns are raised regarding the continued dependence on proprietary commercial LLMs such as Claude 3.5 Sonnet for biomedical annotation. Greater emphasis is suggested on open-source biomedical LLMs, multimodal vision-language models, local deployment, reproducibility, privacy preservation, and regulatory compliance to ensure more transparent, reliable, and sustainable medical annotation systems for translational bioinformatics.

I read with great interest the recently published article by Cheng *et al.* in *Briefings in Bioinformatics* [1]. The authors present an ambitious benchmarking effort across 79 large language models (LLMs) and propose *AICellType* as a practical annotation platform for single-cell and spatial transcriptomics. Their work is timely and valuable, particularly in addressing the limitations of static marker-based annotation systems and introducing semantic reasoning into cell type identification.

I would, however, like to offer a few constructive reflections regarding the continued dependence on proprietary commercial LLMs-especially Claude 3.5 Sonnet-as the primary engine for biological and potentially clinical cell annotation in 2026, when multiple high-performing open-source biomedical LLMs and multimodal vision-language models (VLMs) have become increasingly mature, transparent, and deployable.

First, although the manuscript thoughtfully acknowledges reproducibility concerns associated with proprietary application programming interfaces (APIs), the practical implementation still relies substantially on externally hosted closed models such as Claude, Generative Pre-trained Transformer (GPT), Gemini, Grok, and others from families including Anthropic, OpenAI, Google, xAI, Meta, Alibaba, DeepSeek, Mistral, NVIDIA, and others. This creates an important discussion point between the stated goal of openness and the methodological dependence on systems whose parameters, training data, version changes, and internal optimization processes remain inaccessible to the broader scientific community. In biomedical research-particularly where downstream interpretation may influence translational or diagnostic decisions-greater transparency is highly desirable.

Second, medical and biological annotation often requires domain-specific trustworthiness [2, 3] beyond general linguistic capability. Foundation models such as Claude 3.5 Sonnet may perform strongly in semantic matching, but they are not inherently designed as biomedical reasoning systems. By 2026, several open-source domains-specialized medical LLMs such as Llama3.1-Aloe-Beta-8B, Bio-Medical-Llama-3-8B variants (originated by ZeroOne.AI, FL-finetune-JB-DC, AI4EOSC Team, Gachon Cognitive Computing Lab, mHealth Lab, and ZJUDAI) (https://flower.ai/benchmarks/llm-leaderboard/medical/) and biomedical retrieval-augmented generation [4] systems offer stronger interpretability, local deployment capability, institutional control, and privacy preservation. In addition, pathology-aware multimodal VLMs integrating histology, imaging, and transcriptomic context may provide stronger biological grounding than text-only annotation pipelines.

Third, the study employs a simplified scoring scheme of full match (1), partial match (0.5), and mismatch (0), largely based on ontology proximity. While this is practical and useful for benchmarking, it may sometimes overestimate semantic correctness while underrepresenting the biological importance of certain distinctions. For example, partial matching between immune subtypes may still represent a meaningful difference in oncology, immunotherapy, or infectious disease research. In medical annotation settings, biological relevance and clinical implications may deserve additional emphasis alongside ontological similarity.

Fourth, reliance on OpenRouter (https://openrouter.ai/) and third-party API brokers also raises important considerations related to patient data governance, institutional compliance, and long-term sustainability. Many hospitals, biobanks, and translational laboratories operate under strict privacy regulations, ethics committee requirements, and national biomedical data governance frameworks that may limit the use of external commercial endpoints. In such contexts, local deployment using open-source medical LLMs becomes not only advantageous but often practically necessary [5].

Fifth, the authors describe *AICellType* as ‘open-source,’ and the platform itself is indeed valuable in providing accessible workflows and integration. However, because the central reasoning engine depends primarily on proprietary models, reproducibility may still remain partially constrained. Open science may benefit further when both the interface and the core inference mechanisms are independently inspectable and verifiable.

I believe the future of medical cell annotation may benefit from hybrid systems that combine open biomedical LLMs, domain-specific fine-tuning, retrieval-augmented biological knowledge bases, ontology-constrained reasoning, and multimodal VLM support, alongside carefully selected commercial models where appropriate. Such an approach could strengthen reproducibility, privacy, regulatory compliance, and long-term scientific accountability.

The contribution of Cheng *et al.* is important and deserves appreciation. As the field continues to evolve rapidly, future versions of *AICellType* may further benefit from prioritizing community-controlled open biomedical foundation models alongside proprietary systems, helping the platform mature into an even more robust infrastructure for translational bioinformatics.

**Key Points**

- *AICellType* provides a valuable and timely contribution to large language model (LLM)-based cell type annotation in single-cell and spatial transcriptomics.
- Dependence on proprietary commercial LLMs may limit transparency, reproducibility, and scientific verification in biomedical applications.
- Open-source biomedical LLMs and multimodal vision-language models offer stronger interpretability, privacy control, and local deployment advantages.
- Ontology-based partial matching may not fully capture biologically or clinically significant annotation differences.
- Future platforms may benefit from hybrid systems combining open biomedical models, retrieval-augmented generation, ontology-aware reasoning, and multimodal biological context.

## Data Availability

No data is available.

## Notes

Partha Pratim Ray is an Assistant Professor at Sikkim University, India. His research focuses on agentic artificial intelligence, edge-IoT intelligence, and pervasive biomedical informatics.

## References

[1] Cheng C, Fang S, Zuo Q et al. AICellType: a large language model-based platform for accurate cell type annotation. *Brief Bioinform* 2026;27:bbag151. 10.1093/bib/bbag151 42001469 PMC13092268

[2] Keer L, Liang Z, Pan D et al. Med-R2: Crafting trustworthy LLM physicians via retrieval and reasoning of evidence-based medicine. In: Proceedings of the ACM Web Conference 2026, pp. 1864–75. Dubai, United Arab Emirates: ACM, 2026.

[3] Wang Y, Wang B, Mercer R et al. Trustworthy medical question answering: An evaluation-centric survey. In: Proceedings of the 2025 Conference on Empirical Methods in Natural Language Processing, pp. 27477–90. Suzhou, China: The Association for Computational Linguistics (ACL), 2025.

[4] Abo El-Enen M, Saad S, Nazmy T. A survey on retrieval-augmentation generation (RAG) models for healthcare applications. *Neural Comput Applic* 2025;37:28191–267. 10.1007/s00521-025-11666-9

[5] Xiao J, Li Q, Yang Y et al. Unleashing the power of LLMs for medical video answer localization. In: Gee JC, Alexander DC, Hong J, Iglesias JE, Sudre CH, Venkataraman A, Golland P, Kim JH, Park J (eds.), *International Conference on Medical Image Computing and Computer-Assisted Intervention*, pp. 669–79. Springer Nature Switzerland: Cham, 2025. 10.1007/978-3-032-04981-0_63

